# Using Multivariate Adaptive Regression Splines to Estimate Summed Stress Score on Myocardial Perfusion Scintigraphy in Chinese Women with Type 2 Diabetes: A Comparative Study with Multiple Linear Regression

**DOI:** 10.3390/diagnostics15172270

**Published:** 2025-09-08

**Authors:** Chien-Han Yuan, Po-Chun Lee, Sheng-Tang Wu, Chung-Chi Yang, Ta-Wei Chu, Dong-Feng Yeih

**Affiliations:** 1 Department of Otolaryngology, Kaohsiung Armed Forces General Hospital, Kaohsiung 802, Taiwan; han86449@gmail.com; 2 Institute of Medical Science and Technology, National Sun Yat-sen University, Kaohsiung 804, Taiwan; chyun0124@gmail.com; 3 Department of Otolaryngology, National Defense Medical University, Taipei 114, Taiwan; 4 Medical Education and Research Center, Kaohsiung Armed Forces General Hospital, Kaohsiung 802, Taiwan; 5 Department of Internal Medicine, Kaohsiung Armed Forces General Hospital, Kaohsiung 802, Taiwan; 6 School of Medicine, National Defense Medical University, Taipei 114, Taiwan; 7 Division of Urology, Department of Surgery, Tri-Service General Hospital, National Defense Medical University, Taipei 114, Taiwan; doc20283@gmail.com; 8 Division of Urology, Department of Surgery, Kaohsiung Armed Forces General Hospital, Kaohsiung 802, Taiwan; 9 Division of Cardiovascular Medicine, Taoyuan Armed Forces General Hospital, Taoyuan 325, Taiwan; t220979@gmail.com; 10 Cardiovascular Division, Tri-Service General Hospital, National Defense Medical University, Taipei 114, Taiwan; 11 School of Medicine, National Tsing Hua University, Hsinchu 300, Taiwan; 12 Institute of Bioinformatics and Structural Biology, National Tsing Hua University, Hsinchu 300, Taiwan; 13 Department of Obstetrics and Gynecology, Tri-Service General Hospital, National Defense Medical University, Taipei 114, Taiwan; taweichu@gmail.com; 14 MJ Health Research Foundation, Taipei 114, Taiwan; 15 School of Medicine, College of Medicine, Fu Jen Catholic University, New Taipei City 242, Taiwan; 16 Division of Cardiology, Department of Internal Medicine, Fu Jen Catholic University Hospital, Fu Jen Catholic University, New Taipei City 243, Taiwan

**Keywords:** type 2 diabetes mellitus, multiple adaptive regression spline, myocardial perfusion scan, Shapley addictive explanation

## Abstract

**Background:** Myocardial perfusion scintigraphy (MPS) is an important tool for evaluating ischemia in diabetic populations. However, applications of advanced predictive models like multivariate adaptive regression splines (MARS) to estimate summed stress scores (SSS) are lacking. **Methods:** In this study, 1028 diabetic women undergoing Thallium-201 MPS were analyzed. The dataset was split into training (80%) and testing (20%) subsets. MARS and multiple linear regression (MLR) models were constructed to predict SSS, and their performance was evaluated using root mean square error (RMSE), relative absolute error (RAE), root relative squared error (RRSE), Mean Absolute Percentage Error (MAPE), and Symmetric Mean Absolute Percentage Error (SMAPE). **Results:** On the testing dataset, the MARS model outperformed the MLR model across all metrics, with an RMSE of 3.25 compared to 3.89 for MLR, an RAE of 0.52 vs. 0.64, and an RRSE of 0.53 vs. 0.67. Similar trends were observed in MAPE (18.7% vs. 22.1%) and SMAPE (17.3% vs. 20.5%). **Conclusions:** The superior predictive accuracy of the MARS model suggests its potential to enhance non-invasive myocardial risk stratification in diabetic women.

## 1. Introduction

Type 2 diabetes mellitus (T2DM) is highly prevalent in Taiwan, with approximately 2 million individuals affected. T2DM is associated with comorbidities and places a significant economic burden on healthcare systems. Beyond its significant health consequences, T2DM contributes to severe comorbidities and places a substantial economic burden on society. Previous studies indicate that T2DM is associated with an increased risk of coronary artery disease (CAD), with some reporting an approximately 70% higher risk of acute myocardial infarction in affected individuals compared to the general population [1,2,3]. Schramm et al. reported that individuals with T2DM have a cardiovascular disease (CVD) risk comparable to non-diabetic individuals with a prior myocardial infarction in their study population [2]. CAD in patients with T2DM is often asymptomatic, with some cases at an advanced stage by the time symptoms are detected [4,5]. Consequently, early detection of CAD in this population is essential for timely intervention. Clinical guidelines identify high-risk patients with T2DM for cardiac screening [6,7]. Invasive coronary angiography is a standard method for early CAD diagnosis, but its use is limited by invasiveness and risks. Computed tomography coronary angiography is a non-invasive alternative, though costs and image quality variations due to patient-specific factors may limit its application [8,9]. Exercise electrocardiogram is a non-invasive approach suitable for individuals capable of achieving the required exercise threshold [10,11]. Pharmacologic stress testing using myocardial perfusion scintigraphy (MPS) with Thallium-201 is another non-invasive diagnostic method that has been shown to detect CAD and assess cardiovascular risk in patients with T2DM [12]. For instance, Scholet et al. observed a 37% prevalence of abnormal MPS results in 120 asymptomatic T2DM patients, identifying smoking, T2DM duration, and the total cholesterol/high-density lipoprotein cholesterol (HDL-C) ratio as associated risk factors [13]. Similarly, Prior et al. reported that 31% of 133 T2DM patients exhibited stress-induced perfusion abnormalities on MPS [14]. In a three-year longitudinal study, Nakajima et al. demonstrated that a high SSS on MPS was predictive of worse cardiovascular outcomes [15]. These findings indicate that MPS can identify CAD, and it has been used as a surrogate marker for CAD in research settings [16,17].

Multiple risk factors, including smoking, high-density lipoprotein cholesterol (HDL-C) levels, sex differences, and blood pressure, are associated with CAD [18]. Prior studies have often used conventional statistical techniques. Recent advancements in artificial intelligence have increased the use of machine learning (ML) approaches in medical research. One method, multivariate adaptive regression splines (MARS), models complex, non-linear relationships. Compared to multiple linear regression (MLR), MARS generates interpretable equations to capture variable interactions.

Although attenuation artifacts from breast tissue can reduce the diagnostic accuracy of MPS in women, this study specifically targeted female patients with T2D because of their heightened risk of silent CAD and atypical clinical presentations [2,5]. MPS remains a widely used, non-invasive modality in this population, and the development of a tailored predictive model may aid clinical decision-making in settings lacking advanced cardiac imaging [12,13,15]. Moreover, our cohort was derived from a health screening program consisting solely of female participants, enabling a focused investigation. Nonetheless, attenuation effects and gender-specific variability are acknowledged as limitations in interpreting SSS outcomes, particularly in those with an elevated Body Mass Index (BMI), which has been shown to reduce diagnostic accuracy in MPS [19].

MARS has been applied in clinical research to a limited extent, with studies demonstrating its ability to model non-linear relationships compared to MLR. For example, a study in Turkey applied MARS to predict the body weight of buffalos—a task traditionally requiring specialized equipment—using input variables such as tail length, shoulder height, and withers height, achieving accurate estimations [20].

In addition, this study uses SHapley Additive exPlanations (SHAPs) to interpret the influence of individual features on SSS. The current research has three main objectives: (1) to evaluate whether MARS outperforms MLR in predicting SSS; (2) to construct an equation for estimating SSS; and (3) to use SHAPs to visualize the contribution of each predictor to SSS in a cohort of Taiwanese women.

## 2. Materials and Methods

### 2.1. Participant and Study Design

**Figure 1:**
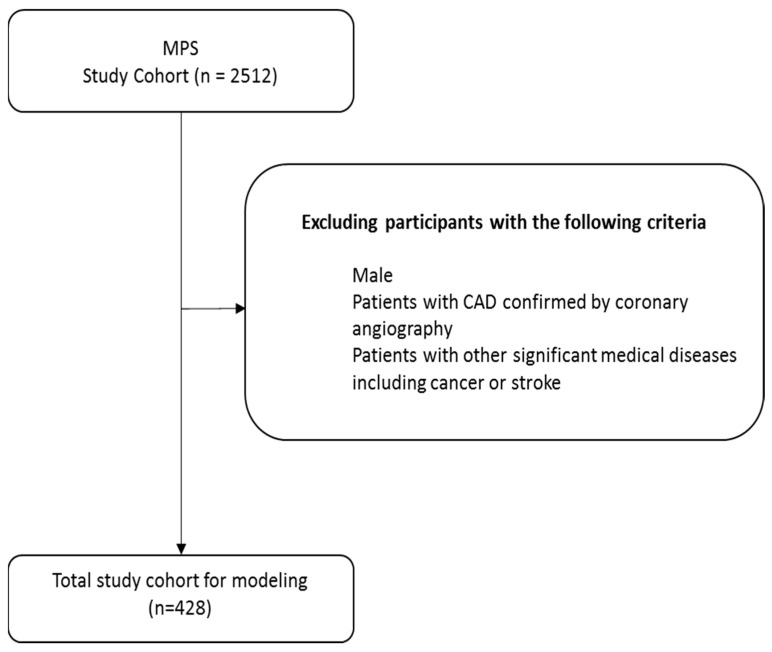
Participant selection.

This study enrolled a total of 428 women patients with T2DM, aged from 30 to 95 years, who had undergone MPS from 2020 to 2024, using data authorized and provided by the MJ Health Research Foundation (Authorization Code: MJHRF2024020A). Informed consent was obtained from all subjects involved in the study. The study protocol received approval from the Institutional Review Board of Kaohsiung Armed Forces General Hospital (IRB No.: KAFGHIRB113-006 and date of approval 2 May 2024). T2DM was diagnosed based on the 2012 American Diabetes Association criteria [21]. The patient selection scheme is shown in Figure 1.

Inclusion criteria:

1. Women with T2DM aged 30–95 years.
2. Hemoglobin A1c (HbA1c) 6.5–10%.
3. BMI 22–30 kg/m^2^.

Exclusion criteria:

1. Patients with confirmed CAD, myocardial infarction, valvular heart disease, or non-ischemic cardiomyopathy.
2. Other significant diseases (e.g., cancer, stroke).

BMI was calculated as weight (kg)/height^2^ (m^2^). Systolic and diastolic blood pressures were measured using a mercury sphygmomanometer. Blood samples were collected for biochemical analysis.

### 2.2. MPS

Pharmacologic stress was induced with dipyridamole (0.56 mg/kg over 4 min), followed by Thallium-201 injection and imaging, as previously reported [22]. The myocardium was segmented into 17 regions, scored on a 5-point scale (0 = normal, 4 = absent uptake) [23]. SSS and summed rest score were calculated, with SSS used as the primary endpoint for CAD detection [15,23,24].

### 2.3. Laboratory Evaluation

Blood samples were collected after a 10 h fast, with plasma stored at −70 °C [22]. Fasting plasma glucose, insulin, and lipid levels were measured using established methods (YSI 203, Yellow Springs, OH, USA; Fuji Dri-Chem 3000, Tokyo, Japan; Bio-Rad Variant II, Hercules, CA, USA, and radioimmunoassay). HOMA-IR was calculated per Matthews et al. [25].

### 2.4. Machine Learning Method

The MARS algorithm was used to model non-linear relationships between clinical variables and the SSS derived from myocardial perfusion scintigraphy, compared against a benchmark MLR model [26,27,28,29,30,31,32,33,34]. MARS was chosen for its ability to capture complex, non-linear interactions and higher-order relationships among predictors, making it suitable for high-dimensional clinical data [35]. The dataset was randomly split into 80% training and 20% testing subsets. The training subset was further divided for hyperparameter tuning, using a grid search to optimize MARS parameters (e.g., maximum degree of interaction, number of basis functions, and knot locations) to minimize root mean squared error (RMSE).

Feature selection was performed using recursive feature elimination (RFE) within the training phase to identify the most predictive variables (e.g., HbA1c, HOMA-IR, LDL-C, and SBP) for SSS. Cross-validation (10-fold) was applied to assess model stability and prevent overfitting. The final MARS model was evaluated on the test set using multiple regression metrics: RMSE, relative absolute error (RAE), root relative squared error (RRSE), Mean Absolute Percentage Error (MAPE), and Symmetric Mean Absolute Percentage Error (SMAPE), used to evaluate MLR and MARS models (Table 1). SHAPs were used to quantify feature importance and interpret the contribution of each predictor to SSS predictions, visualized through summary and waterfall plots [36].

To enhance clinical applicability, the MARS model was translated into an explicit equation embedded in an Excel tool, leveraging the MAX function to handle hinge functions inherent to MARS. Comparative analysis with MLR was conducted on the same dataset, with MARS consistently demonstrating lower error rates across all metrics, indicating superior predictive performance. No formal hypothesis testing (e.g., paired *t*-test) was applied due to the cross-sectional design, but the consistent error reduction supports MARS’s robustness [35].

Analyses were performed using R (version 4.0.5, packages: earth 5.3.3, caret 6.0–94, and stats) and Python (v3.9.13; packages: SHAP v0.42.1, pandas v1.5.3, Numby v1.24.4, and Matplotlib v3.7.1) for SHAP visualizations [36,37].

### 2.5. Statistical Analysis

Data normality and variance were assessed using Kolmogorov–Smirnov and Levene’s tests. Continuous variables were reported as mean ± SD. The *t*-tests compared smokers vs. non-smokers, and Pearson correlation analyzed clinical parameters and SSS. MLR served as the benchmark for MARS comparisons. Analyses were performed using SPSS 13.0, with *p* < 0.05 indicating significance.

In summary, to statistically differentiate the performance of the MARS and MLR models, we first evaluated both using identical training and testing datasets to ensure a fair comparison. Five standard regression metrics, RMSE, RAE, RRSE, MAPE, and SMAPE, were calculated on the testing set. We then directly compared these error values across the models to assess performance. While a formal hypothesis test (e.g., paired *t*-test or bootstrapping on error residuals) was not applied due to the cross-sectional design and the ML work, the consistent pattern of lower error rates across all metrics supports the robustness of the MARS model’s improved performance. This approach is commonly used in the predictive modeling literature when comparing algorithmic estimators on fixed test sets [35]. Furthermore, SHAPs were applied to the final MARS model to examine feature-level contributions, offering a layer of interpretability rather than additional model validation.

## 3. Results

**Figure 2:**
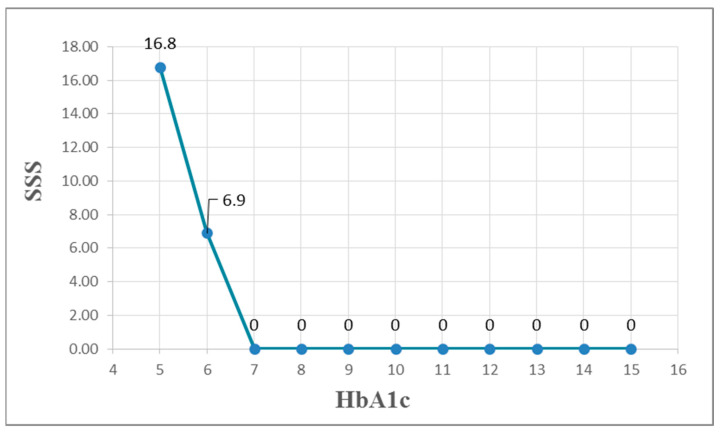
The non-linear relationships between HbA1c and SSS of MPS. Note: HbA1c: hemoglobin A1c; SSS: sum stress score; and MPS: myocardial perfusion scintigraphy.

**Figure 3:**
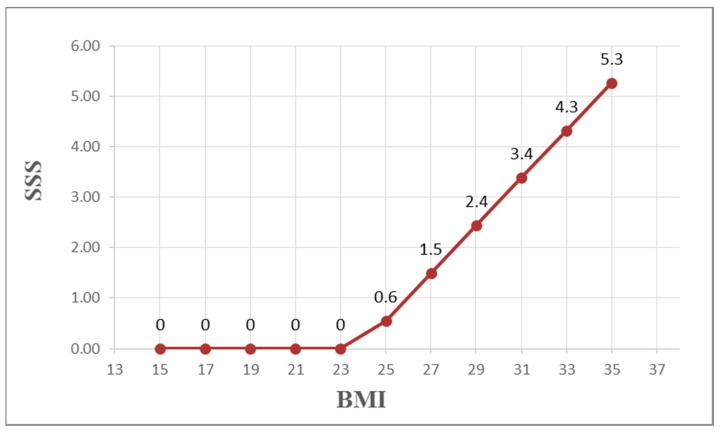
The non-linear relationships between BMI and SSS of MPS. Note: BMI: Body Mass Index; SSS: sum stress score; and MPS: myocardial perfusion scintigraphy.

**Figure 4:**
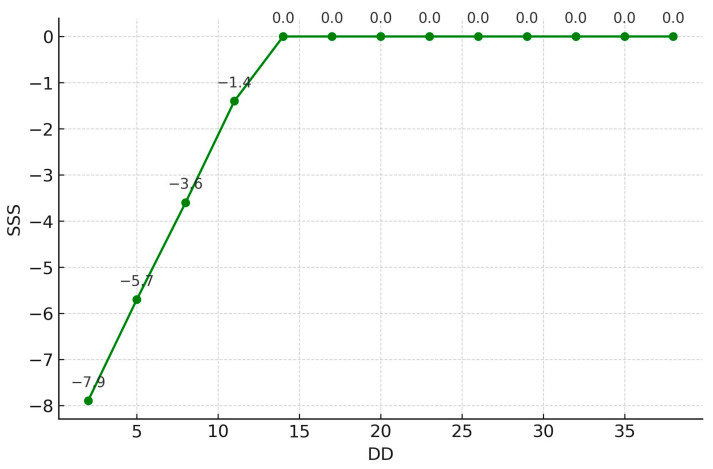
The non-linear relationships between DD and SSS of MPS. Note: DD: duration of diabetes; SSS: sum stress score; and MPS: myocardial perfusion scintigraphy.

Demographic and biochemical data and SSS results are presented in Table 2. No significant difference in SSS was observed between smokers and non-smokers (*p* = 0.677). Simple correlations between clinical parameters and SSS are presented in Table 3. BMI was positively correlated with the microalbumin/creatinine ratio (MCR) (r = 0.261, *p* < 0.001; r = 0.121, *p* = 0.04, respectively) and negatively correlated with HDL-C (r = −0.144, *p* = 0.018). Table 4 shows estimation errors for MLR and MARS models predicting SSS in Taiwanese women with T2DM. For MARS, RAE was 1.0965, RRSE was 1.1883, and RMSE was 8.0443. For MLR, RAE was 1.2073, RRSE was 1.2611, and RMSE was 8.5376. MARS had lower errors than MLR in this study. MARS identified three basis functions: BMI, duration of diabetes (DD), and HbA1c. The resulting equation is presented below. Figure 2, Figure 3 and Figure 4 illustrate the relationships between these basis functions and SSS. We also display the equations by using a Word file, which could be directly copy-pasted to an Excel file, and the estimation of the SSS would be derived (Table 5):

$$SSS=3.064+0.472MAX\left(0,BMI-23.83\right)-0.718MAX\left(0,12-DD\right)\;+9.88MAX\left(0,6.7-HbA1c\right)$$

**Figure 5:**
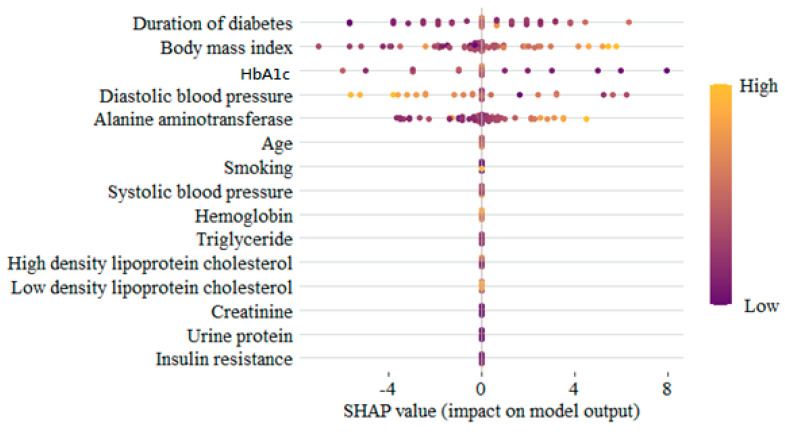
The Bee swarm figure derived from SHAPs.

**Figure 6:**
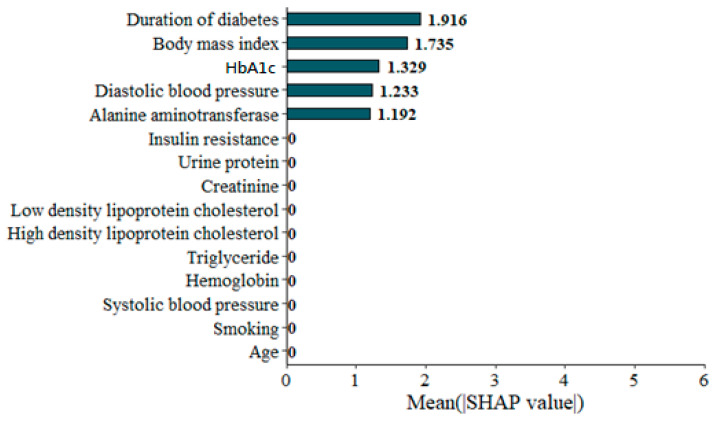
The absolute Shapley addictive explanation of each feature.

As mentioned in the Methods section, the main purpose of SHAPs is to show the contribution value to each variable. At the same time, SHAPs could also show the direction of each variable. In Figure 5, the Bee swarm figure gives a general idea of the contribution of all the features of each participant. On the top, the DD is the most important, followed by BMI, GA, DBP, and ALT. Each dot represents each participant’s contribution to the SSS, with the yellow color indicating higher importance. To show the ‘absolute’ contribution of each feature, the SHAP values are shown in Figure 6. Not surprisingly, the orders of importance are the same as in Figure 6.

## 4. Discussion

We developed a predictive model for SSS in Taiwanese women with T2DM using MARS. This model outperformed traditional MLR, identifying HbA1c, DD, and BMI as the most relevant features.

MPS served as our outcome tool. While not as definitive as coronary angiography, positron emission tomography (PET), or cardiac magnetic resonance imaging (MRI), MPS is widely adopted in clinical practice due to its cost-effectiveness, safety, and accessibility. Its prognostic utility in risk stratification and guiding clinical decisions is particularly beneficial in diabetic populations, who often present with atypical or silent symptoms [15,23].

Among the key predictors, HbA1c represents chronic glycemic burden. Poor glucose control promotes microvascular damage and contributes to myocardial ischemia. The mechanistic pathways linking hyperglycemia to adverse cardiac outcomes include increased oxidative stress, endothelial dysfunction, and the formation of advanced glycation end-products, which impair myocardial perfusion [38,39,40].

The duration of diabetes reflects cumulative metabolic stress. Longer disease duration exacerbates cardiovascular risk not only through persistent hyperglycemia but also via progressive vascular remodeling and autonomic dysfunction. These mechanisms may explain the observed association with higher SSS [41,42,43].

BMI, an established marker of adiposity, is implicated in insulin resistance and systemic inflammation. Excess adipose tissue promotes atherogenesis and impairs myocardial oxygen delivery. The role of BMI in cardiovascular imaging outcomes supports the need for metabolic risk control in this population [44,45,46,47].

Interestingly, the ranking of predictors differed between the MARS model and SHAPs. While MARS aims to optimize prediction, SHAPs provide insight into feature contributions across all predictions. The discrepancy reinforces the complementary nature of modeling and interpretability tools, particularly in clinical applications where both accuracy and explainability are crucial.

The present study provides a preliminary framework for estimating SSS using structured clinical data. This approach offers an interpretable, equation-based model that could be integrated into clinical systems or electronic health records to support decision-making. It may be especially helpful in situations with limited imaging resources or as a triage tool for risk prioritization.

These results are consistent with evolving recommendations in cardiovascular risk assessment guidelines, which promote the use of non-invasive and personalized strategies, particularly in asymptomatic or high-risk populations. This is reflected in the 2010 ACCF/AHA guidelines, which emphasize individualized cardiovascular risk evaluations in asymptomatic adults [48]. Similarly, the 2012 ACCF/AHA stable ischemic heart disease guidelines endorse a risk-based triage, advocating for the selective use of imaging based on pre-test probability [49]. These principles are further supported by prior evidence emphasizing the cost-effective deployment of MPS in populations stratified by clinical characteristics [50].

Furthermore, by demonstrating the utility of routine clinical variables to estimate myocardial perfusion outcomes, this study underscores the potential for scalable, population-level screening approaches. Integrating such predictive models into primary care workflows could enhance the early detection of subclinical cardiovascular risk, streamline resource allocation, and prioritize high-risk individuals for further evaluation. These findings align with the growing emphasis in clinical guidelines on individualized, risk-based screening strategies for coronary artery disease in diabetic patients. By leveraging routinely collected data, this model may support guideline-directed care by identifying those who could benefit from further testing while avoiding unnecessary imaging in low-risk individuals. This model also supports a cost-saving strategy by reducing unnecessary imaging and focusing specialist resources on those most likely to benefit from further diagnostic workups.

This study has several limitations that should be acknowledged. First, the cohort consists exclusively of Chinese women with T2D, which limits the generalizability of the findings to other ethnicities, men, or broader populations. While this homogeneity allows for a focused investigation on a high-risk group often underrepresented in cardiovascular research, it also introduces gender-specific variability in myocardial perfusion imaging, such as potential attenuation artifacts from breast tissue, which may affect the accuracy of SSS quantification, particularly in individuals with a higher BMI [19]. Second, the cross-sectional design precludes causal inferences and limits our ability to assess temporal relationships between risk factors and disease progression. Third, although MPS is a well-validated tool for risk stratification, it has inherent limitations: it may fail to detect balanced ischemia in multivessel coronary artery disease, where globally reduced perfusion can appear “normal” due to uniform tracer uptake. While we excluded patients with known CAD to reduce confounding, the absence of a gold standard diagnostic test—such as invasive coronary angiography, coronary CT angiography, or cardiac PET—for the anatomical or physiological confirmation of coronary stenosis remains a significant constraint in fully validating the SSS as a surrogate endpoint [16,17]. Fourth, some important behavioral and socioeconomic factors were not available in the dataset. Finally, the use of conventional BMI cutoffs may not fully capture adiposity-related risks in this Asian population, and ethnicity-specific thresholds should be considered in future applications. Despite these limitations, the model provides a practical, interpretable tool for preliminary risk estimation using routinely collected clinical data.

## 5. Conclusions

This study developed a predictive equation to estimate the summed stress score of myocardial perfusion scans in Chinese women with type 2 diabetes mellitus. In addition, the model offers a cost-effective alternative for preliminary cardiac risk stratification, especially in clinical environments where access to advanced imaging modalities is limited or not routinely available. The most influential variables were blood sugar control as reflected by glycated hemoglobin, the number of years since diabetes diagnosis, and excess body weight. The results provide a non-invasive, interpretable method to estimate cardiac risk. Although this model does not replace imaging, it offers a practical option for preliminary screening and supports clinical decision-making in resource-limited settings. Feature importance differed slightly when evaluated through SHapley Additive exPlanations, emphasizing the complementary strengths of model prediction and interpretability.

## Figures and Tables

**Table 1:** The equations for estimation errors between MLR and MARS.

Metrics	Description	Calculation
MAPE	Mean Absolute Percentage Error	$MAPE=\frac{1}{n}\sum_{i=1}^{n}\left|\frac{y_{i}-{\hat{y}}_{i}}{y_{i}}\right|\times 100$
SMAPE	Symmetric Mean Absolute Percentage Error	$SMAPE=\frac{1}{n}\sum_{i=1}^{n}\frac{\left|y_{i}-{\hat{y}}_{i}\right|}{\left(\left|y_{i}\right|+\left|{\hat{y}}_{i}\right|\right)/2}\times 100$
RAE	Relative Absolute Error	$RAE=\sqrt{\frac{\sum_{i=1}^{n}{\left(y_{i}-{\hat{y}}_{i}\right)}^{2}}{\sum_{i=1}^{n}{\left(y_{i}\right)}^{2}}}$
RRSE	Root Relative Squared Error	$RRSE=\sqrt{\frac{\sum_{i=1}^{n}{\left(y_{i}-{\hat{y}}_{i}\right)}^{2}}{\sum_{i=1}^{n}{\left(y_{i}-{\hat{y}}_{i}\right)}^{2}}}$
RMSE	Root Mean Squared Error	$RMSE=\sqrt{\frac{1}{n}\sum_{i=1}^{n}{\left(y_{i}-{\hat{y}}_{i}\right)}^{2}}$

**Table 2:** Demographic, biochemistry, and the SSS of MPS.

Numeric Variables	Unit	Mean ± SD
Age	year	69.32 ± 9.64
Body Mass Index	kg/m^2^	26.54 ± 4.43
Duration of Diabetes	year	14.02 ± 8.31
Systolic Blood Pressure	mmHg	131.88 ± 15.16
Diastolic Blood Pressure	mmHg	72.07 ± 10.24
Hemoglobin	g/dL	12.19 ± 1.38
Hemoglobin A1c	%	7.66 ± 1.37
Triglycerides	mg/dL	124.68 ± 74.08
High-Density Lipoprotein Cholesterol	mg/dL	52.74 ± 14.59
Low-Density Lipoprotein Cholesterol	mg/dL	94.01 ± 23.75
Alanine Aminotransferase	IU/L	21.53 ± 11.34
Creatinine	mg/dL	1.02 ± 0.90
Urine protein	mg/L	180.36 ± 630.39
Homeostasis Model Assessment of Insulin Resistance	—	6.26 ± 7.12
Homeostasis Model Assessment of β-cell function	—	100.29 ± 322.20
**Dependent Variable**	**Unit**	**Mean ± SD**
Sum of Stress Score (SSS)	—	4.56 ± 6.74
**Ordinal Variables**	***n* (%)**	***p*-Value**
Smoking Status		
Smoking	10 (6.7%)	0.677
Non-Smoking	140 (93.3%)	

Note: SSS: sum of stress score; MPS: myocardial perfusion scintigraphy.

**Table 3:** Results of Pearson’s correlation between the independent variables and SSS of MPS.

**Variable**	**Age**	**BMI**	**DD**	**SBP**	**DBP**	**Hb**	**HbA1c**	**TG**
SSS	−0.007	0.261 **	−0.001	−0.027	−0.117	−0.054	−0.019	0.032
**Variable**	**HDL-C**	**LDL-C**	**ALT**	**Cr**	**MCR**	**HOMA-IR**	**HOMA-β**	
SSS	−0.144 *	0.008	−0.012	0.032	0.121 *	0.117	−0.007	

Note: BMI: Body Mass Index; DD: duration of diabetes; SBP: Systolic Blood Pressure; DBP: Diastolic Blood Pressure; Hb: hemoglobin; HbA1c: hemoglobin A1c; TG: Triglycerides; HDL-C: High-Density Lipoprotein Cholesterol; LDL-C: Low-Density Lipoprotein Cholesterol; ALT: Alanine Aminotransferase; Cr: creatinine; MCR: microalbumin/creatinine ratio; HOMA-IR: Homeostasis Model Assessment of Insulin Resistance; and HOMA-β: Homeostasis Model Assessment of β-cell function. * *p* < 0.05; **, *p* < 0.01.

**Table 4:** Estimation errors for MLR and MARS.

	RAE	RRSE	RMSE
MARS	1.0965	1.1883	8.0443
MLR	1.2073	1.2611	8.5376

Note: RAE: relative absolute error; RRSE: root relative squared error; RMSE: root mean squared error; MARS: multiple adaptive regression spline; and MLR: multiple linear regression.

**Table 5:** Equation details which could be copied to Excel for estimating SSS of MPS.

	A	B	C
1	Type BMI	=MAX(0, A1 − 23.83)	=0.472 × B1
2	Type diabetic duration	=MAX(0, 12 − A2)	=−0.718 × B2
3	Type hemoglobin A1c	=MAX(6.7 − A3)	=9.88 × B3
4			
5	SSS		
6	=3.064 + C1 + C2 + C3		

Note: SSS: sum stress score; MPS: myocardial perfusion scintigraphy.

## Data Availability

Data available on request due to privacy/ethical restrictions. This study used secondary databases for analysis. The source of the database was from the MJ Health Research Foundation.
